# Supplementary figures and images for: Mechanical stiffness of reconstituted actin patches correlates tightly with endocytosis efficiency
Source: PLoS Biol. 2019 Oct 25;17(10):e3000500. doi: 10.1371/journal.pbio.3000500 (PMC6834286; doi:10.1371/journal.pbio.3000500)

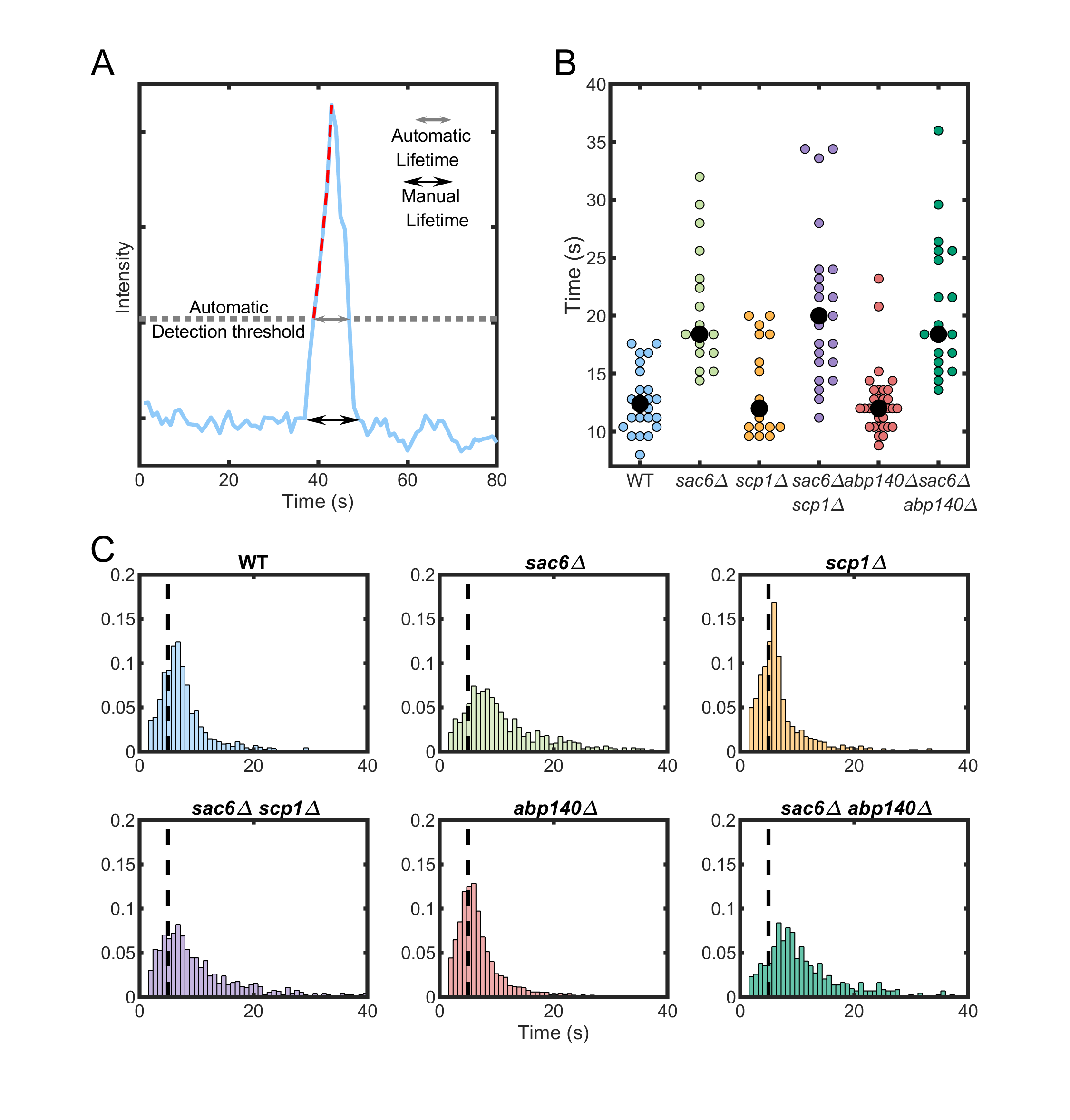

Supplement: S1 Fig — (A) Example of the intensity of an actin patch over time. The manual and automatic tracking lifetimes are indicated with a black arrow and gray arrow, respectively. The higher threshold on detection explains the shorter lifetime measured with TrackMate. The slope of the red dashed line represents the rate of actin assembly. (B) Manual tracking of patch lifetime in the different strains. Each colored dot represents a patch. Black dots indicate the median. (C) Patch lifetime distribution. Patches with a lifetime lower than 5 seconds (dashed line) were not taken into account to avoid detection artifacts. (TIF) [file pbio.3000500.s001.tif]

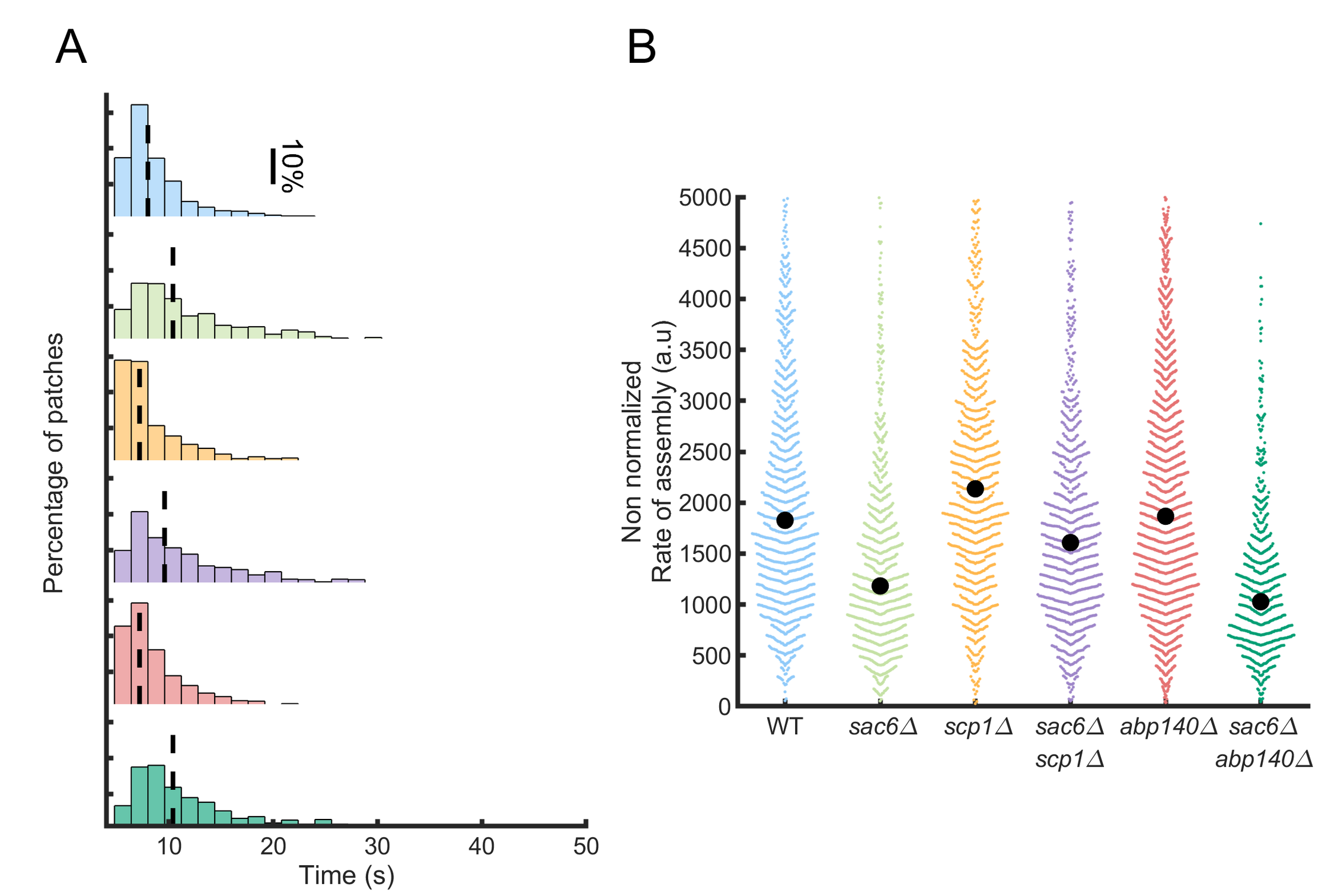

Supplement: S2 Fig — (A) Histogram of patch lifetime extracted from the analysis for the different strains. The median (represented by a dashed line) is 8.0 seconds for WT, 7.2 seconds for scp1Δ and abp140Δ, 10.4 seconds for sac6Δ and sac6Δ abp140Δ, and 9.6 seconds for sac6Δ scp1Δ. The data are pooled from several replicates and non-normalized. (B) Rate of actin patch assembly in the cells. The rates are obtained from the curves of the fluorescence intensity as a function of time during the phase of patch assembly for all strains used in this study. Each colored dot is a measured patch. The median (represented by a black dot) is 1,800 a.u. for WT, 2,100 a.u. for scp1Δ, 1,900 a.u. for abp140Δ, 1,200 a.u. for sac6Δ, 1,000 a.u. for sac6Δ abp140Δ, and 1,600 a.u. for sac6Δ scp1Δ. The data are pooled from several replicates and non-normalized. Abp140, actin-binding protein 140; a.u., arbitrary unit; Sac6, yeast homolog of fimbrin; Scp1, yeast homolog of calponin; WT, wild type. (TIF) [file pbio.3000500.s002.tif]
