# Supplementary material for: Mechanical stiffness of reconstituted actin patches correlates tightly with endocytosis efficiency
Source: PLoS Biol. 2019 Oct 25;17(10):e3000500. doi: 10.1371/journal.pbio.3000500 (PMC6834286; doi:10.1371/journal.pbio.3000500)
Supplement: Attachment — Submitted filename: report.docx [file pbio.3000500.s006.docx]

The manuscript focuses on clatherin mediated endocytosis in yeast. The paper considers 3 putative actin crosslinkers that have been localized to actin patches in yeast, namely, Sac6, Scp1 and Abp140, and compares the dynamics and distribution of actin patches in wild type yeast strains and strains mutated in one or two of these proteins. The paper further studies the assembly dynamics and mechanical properties of actin networks assembled from cell extract generated from the various mutant strains compared to wild type cell extracts and a purified protein system. The ability to compare in vivo phenotypes in various mutants to the behavior of cell extract from these strain in an in vitro assay, as well as to purified proteins provide an important tool to dissect the dynamics. The authors show that Sac6 has a large contribution to the stiffness of actin networks and that this is correlated with enhanced endocytosis.

The combination of in vivo measurements of patch dynamics and distribution in wild type and mutant strains, with in vitro work on cell extracts from these different strains, and in vitro work with purified protein is laudable. In particular, the add back of Sac6 to the mutant strain extract and purified protein system and the measured impact on mechanical stiffness is very illuminating. Overall I think this is a nice contribution to the field, and I recommend accepting this manuscript for publication after the authors address several points (see below) and clarify some points regarding the presentation of the data.

Major comments:

- The first results section is called **“Three putative actin crosslinkers impact differently force generation during clathrin-mediated endocytosis” –** However the data in this section is related to the dynamics of actin patches and contain no actual data on force generation. Please change the title of this section to fit its content.
- The plot in Fig 1A is unclear. What are the axes of the graph? The text indicates: “the fluorescence intensity of the Abp1-GFP actin reporter over time (Fig 1A)”, but the graph has no axes label and a spatial scale for the x-axis.
- The authors refer to “maximum displacements from the membrane”- and “maximal distance travelled”. It is not clear if these are the same, and how they are measured. In particular, it is unclear if the authors refer to displacement within the plane of the membrane or in a perpendicular direction. It is unclear what is the meaning of this measure (i.e. what are the implications of larger/smaller displacement of the patches within the membrane).
- Related question- the authors use the criteria that maximal displacement >0.2um for measuring the “internalization efficiency”. Can the authors explain why these two quantities are at all related? What is the rationale?
- The measurements of actin assembly time (lines 91-195) are reported- where is the data? Specifically, it is unclear if these are the same measurement as the life time (as shown in Fig S1B) or are they different?
- The authors explain the faster growth of actin in Sac6 mutant extract, and the slower growth of actin patches in these strains in vivo by differences in the polmer-monomer ratio in the extracts. This sounds plausible – but it will depend very strongly on what actin populations are probed in the cell extracts. Can the authors elaborate on this- do they think that the extract retains all the actin from the cell (polymer+monomer)? Or does the extract contain only the cytosolic fraction? Is there any endogenous actin nucleation activity in the extracts?
- The authors indicate that “As the patch lifetime and the rate of actin assembly significantly differed

between replicates, we normalized them by the median of the WT observed on the

same day, pooled the normalized data of the different strains …. “ . The authors should provide the non-normalized data (at least in a supplementary figure) and present the distribution of all the data without normalization and discuss the meaning of their results given this variability. It is not accetable to “hide” the experimental variability in this manner.

- Is it possible to examine if the influence of Sac6 on the mechanics of the network in vitro are the same whether it is added before the networks are assembled, or added to the system after the networks reach a plateau?

Two more conceptual comments:

- The authors attribute the effect of Sac6 to the mehcanics to its crosslinking activities. It is unclear to me why adding crosslinkers to a patch of short and branched filaments in an Arp2/3 generated will have such a dramatic effect on the mechanics. Naively, I would think that the branches are stiffer than crosslinks, and that such short filaments are also rather stiff. Could it be that the protein is a filament binding protein which stiffen the mechanical properties of filaments rather than crosslinks? Is there a way to test crosslinking vs stiffening individual filaments? It is also interesting to note in this context that the concentration of Sac6 used with the purified proteins reaches nearly the density of actin monomers used.
- Regarding the simple model that the authors bring in the section “**Relation between patch rigidity and endocytosis efficiency”.** I am not sure how helpful it is- the force on the actin network depends on the boundary condition near the membrane (which would depend on the turgor pressure), but the system likely has a force-free boundary condition on its cytoplasmic side. Thus the actual stress within the network will be highly dependent on geometry. I am not sure if thinking of a slab of network with the turgor pressure as the opposing stress is relevant. If the authors feel this discussion is useful they should better explain the relevant physics- given both boundary condition at both side of the network and the geometry of the network.

Minor comments:

- Ref. 22- there is no journal name
- Lines 291-292 “This is however IS not very surprising…”
