# Supplementary material for: Mechanical stiffness of reconstituted actin patches correlates tightly with endocytosis efficiency
Source: PLoS Biol. 2019 Oct 25;17(10):e3000500. doi: 10.1371/journal.pbio.3000500 (PMC6834286; doi:10.1371/journal.pbio.3000500)
Supplement: Attachment — Submitted filename: Reviewers comments final.docx [file pbio.3000500.s007.docx]

**To Editorial Board PLoS Biology	September 26^th^, 2019**

**Manuscript** PBIOLOGY-D-19-02292R1

**Response to the comments of the editor and the reviewers**

Dear Editor and Reviewers,

We would like to thank you for your excellent comments and suggestions on our work. We have now revised our manuscript according to your comments. We feel that the requested modifications improve the manuscript.

Please find below (text in purple) a point-by-point response to all of your comments that indicates the changes we have made to the manuscript.

Sincerely,

**Olivia du Roure** (ESPCI Paris), **Alphée Michelot** (IBDM Marseille) and **Julien Heuvingh** (ESPCI / Univ Paris)

**Reviewer #1’s comments:**

The manuscript focuses on clatherin mediated endocytosis in yeast. The paper considers 3 putative actin crosslinkers that have been localized to actin patches in yeast, namely, Sac6, Scp1 and Abp140, and compares the dynamics and distribution of actin patches in wild type yeast strains and strains mutated in one or two of these proteins. The paper further studies the assembly dynamics and mechanical properties of actin networks assembled from cell extract generated from the various mutant strains compared to wild type cell extracts and a purified protein system. The ability to compare in vivo phenotypes in various mutants to the behavior of cell extract from these strain in an in vitro assay, as well as to purified proteins provide an important tool to dissect the dynamics. The authors show that Sac6 has a large contribution to the stiffness of actin networks and that this is correlated with enhanced endocytosis.

The combination of in vivo measurements of patch dynamics and distribution in wild type and mutant strains, with in vitro work on cell extracts from these different strains, and in vitro work with purified protein is laudable. In particular, the add back of Sac6 to the mutant strain extract and purified protein system and the measured impact on mechanical stiffness is very illuminating. Overall I think this is a nice contribution to the field, and I recommend accepting this manuscript for publication after the authors address several points (see below) and clarify some points regarding the presentation of the data.

We thank Reviewer #1 for his/her assessment of this manuscript and the suggested clarifications he/she suggested.

Major comments:

- The first results section is called **“Three putative actin crosslinkers impact differently force generation during clathrin-mediated endocytosis” –** However the data in this section is related to the dynamics of actin patches and contain no actual data on force generation. Please change the title of this section to fit its content.

We have modified the title of this section for **“Three putative actin crosslinkers impact differently membrane invagination during clathrin-mediated endocytosis”**

- The plot in Fig 1A is unclear. What are the axes of the graph? The text indicates: “the fluorescence intensity of the Abp1-GFP actin reporter over time (Fig 1A)”, but the graph has no axes label and a spatial scale for the x-axis.

We modified the text of the caption to be clearer about this plot (“The plasma membrane is represented by the horizontal bold line tangent to the X axis while the Y-axis represents the normal to the membrane and is oriented towards the interior of the cell. Identical scales have been used for the two axes.”). We also added a grid to facilitate the reading of the figure.

- The authors refer to “maximum displacements from the membrane”- and “maximal distance travelled”. It is not clear if these are the same, and how they are measured. In particular, it is unclear if the authors refer to displacement within the plane of the membrane or in a perpendicular direction. It is unclear what is the meaning of this measure (i.e. what are the implications of larger/smaller displacement of the patches within the membrane).

We apologize for the confusion. In the new version of the manuscript, we only refer to the term “Maximum patch displacement” and provide clear explanations. What is measured is the distance between the first point of the trajectory and the furthest point reached by the patch inside the cell. Observations of patch movements are made in the medial focal plane of the cells, which is orthogonal to the plane of the membrane. Therefore, actin patch movements are not observed necessarily only in the plane of the membrane, but also towards the interior of the cell.

- Related question- the authors use the criteria that maximal displacement >0.2um for measuring the “internalization efficiency”. Can the authors explain why these two quantities are at all related? What is the rationale?

A distance of 0.2 µm is typically chosen in studies of endocytosis as a distance above which an endocytic event has successfully occurred. It is used in other studies such as Kaksonen et al., *Cell*, 2005 or Kukulski et al., *Cell*, 2012. We clarify this point in the text.

- The measurements of actin assembly time (lines 91-195) are reported- where is the data? Specifically, it is unclear if these are the same measurement as the life time (as shown in Fig S1B) or are they different?

We apologize for the confusion. We distinguish the patch lifetime, expressed in seconds, which appeared in S1A Fig (now S1B Fig), from the rate of patch assembly, expressed in arbitrary unit per second. This rate is measured from the same data set as the one used for the life time. We measured the intensity of Abp1-GFP over time. We reported the slope of the curve of intensity vs time during the assembly phase. Missing details of the description of these measurements were added in the Result section and in the Material and Methods. We also modified S1 Fig for a better understanding.

- The authors explain the faster growth of actin in Sac6 mutant extract, and the slower growth of actin patches in these strains in vivo by differences in the polmer-monomer ratio in the extracts. This sounds plausible – but it will depend very strongly on what actin populations are probed in the cell extracts. Can the authors elaborate on this- do they think that the extract retains all the actin from the cell (polymer+monomer)? Or does the extract contain only the cytosolic fraction? Is there any endogenous actin nucleation activity in the extracts?

Those are very good questions which pertain to all studies performed with cellular extracts. We have elaborated on that in the text (page 10).

Actin polymer/monomer (F/G) ratio:

As described in this manuscript and previous manuscripts (Michelot et al., *Current Biology*, 2010; Michelot and Drubin, *Methods in enzymology*, 2014), yeast extracts are prepared by freezing yeast cells in liquid nitrogen, grinding them, thawing them with buffer and protease inhibitors and a centrifugation step to remove insoluble components. We know that a large fraction of the actin remains present in the extracts, but we cannot tell whether the F/G actin ratio is maintained as it is in cells during this procedure. Also we believe that cellular extracts behave like any biochemical system and reach after their preparation a steady-state equilibrium with a given G/F actin ratio which should not depend on the presence of crosslinkers. However, how this ratio compares to the cellular ratio is quite difficult to assess.

Endogenous actin nucleation activity:

Some actin nucleators surely remain in the extracts and are active (see Michelot et al., *Current Biology*, 2010). But their concentration is not high enough to deplete the monomeric actin pool as large amounts of actin do assemble on beads rapidly. In the case of this specific study, we also do not see why endogenous actin nucleation activity would account for any differences between extracts tested in this work, where only the actin crosslinking activities are perturbed.

- The authors indicate that “As the patch lifetime and the rate of actin assembly significantly differed between replicates, we normalized them by the median of the WT observed on the same day, pooled the normalized data of the different strains …. “ . The authors should provide the non-normalized data (at least in a supplementary figure) and present the distribution of all the data without normalization and discuss the meaning of their results given this variability. It is not accetable to “hide” the experimental variability in this manner.

As requested by Reviewer #1, we provide in the revised manuscript the non-normalized data in S2 Fig for both the patch lifetime and the rate of actin assembly. Small variations on different days of experiments is classical as endocytosis is very sensitive to parameters such as temperature or food. As the automatic method we used to measure these quantities provide a large number of points for each replicate, a significant variability between replicates is not surprising. However, normalizing the data to the behavior of wild-type cells allows reducing significantly the systematic error between replicates.

- Is it possible to examine if the influence of Sac6 on the mechanics of the network in vitro are the same whether it is added before the networks are assembled, or added to the system after the networks reach a plateau?

In the experiments performed from wild-type extracts, as it is the case in cells, Sac6 is systematically present when actin networks assemble. For this reason, Sac6 was added to the cell extract or with the purified proteins from the onset of polymerization for a straightforward comparison of the results.

It is technically possible to add Sac6 after the polymerization of the actin networks around the beads. However this configuration may also lead to an uneven coating of actin networks with the crosslinkers. Given the mesh size of the network and the high affinity of Sac6 for actin filaments, it could create a crust of crosslinked actin filaments at the outer periphery of the networks. Although inhomogeneous actin networks are certainly interesting to study, they are more difficult to analyze and we think that this experiment would be beyond the scope of this study.

Two more conceptual comments:

- The authors attribute the effect of Sac6 to the mehcanics to its crosslinking activities. It is unclear to me why adding crosslinkers to a patch of short and branched filaments in an Arp2/3 generated will have such a dramatic effect on the mechanics. Naively, I would think that the branches are stiffer than crosslinks, and that such short filaments are also rather stiff. Could it be that the protein is a filament binding protein which stiffen the mechanical properties of filaments rather than crosslinks? Is there a way to test crosslinking vs stiffening individual filaments? It is also interesting to note in this context that the concentration of Sac6 used with the purified proteins reaches nearly the density of actin monomers used.

We agree with the referee that the flexibility of the crosslinking proteins might be larger than the flexibility of actin filament. However at the scale the network other effects of the crosslinkers can affect the mechanics.

- The first one is the connectivity. In endocytic patches, actin filaments are branched by the Arp2/3 complex and as branches only connect three strands of filaments, the number of mechanical constraints in a branched network is rather small. This number may place Arp2/3 actin networks close to the isostatic point below which the network cannot efficiently resist deformation: one can illustrate this by the fact very few paths of filaments connect both ends of the network. Adding crosslinkers**, which connect four strands**, increases efficiently this connectivity. As more and more crosslinks are introduced, the network is more and more connected, getting away from the isostatic point and as a result get stiffer. For recent references about this phenomenon, see Broedersz Nature Physics 2011 and Merkel PNAS 2019.
- The second one is the reduction of the distance between contact points between filaments. Adding crosslinkers adds geometrical constraints to the deformation of the filament. The relevant length on which the filaments can bend is thus reduced in presence of crosslinkers (divided by two if the filament is crosslinked at its middle point.) As the force required to bend an elastic filament depends linearly on the inverse of its length, it will impact severely the mechanics of the network.

Testing between crosslinking and stiffening is difficult in the case of fimbrin. Indeed, this protein interacts with single actin filaments with a much weaker affinity than for actin bundles (Christensen et al., *eLife*, 2017 and our own data). As for numerous other actin crosslinkers, it is the simultaneous interaction with two filaments that reinforces the interaction with actin.

- Regarding the simple model that the authors bring in the section “**Relation between patch rigidity and endocytosis efficiency”.** I am not sure how helpful it is- the force on the actin network depends on the boundary condition near the membrane (which would depend on the turgor pressure), but the system likely has a force-free boundary condition on its cytoplasmic side. Thus the actual stress within the network will be highly dependent on geometry. I am not sure if thinking of a slab of network with the turgor pressure as the opposing stress is relevant. If the authors feel this discussion is useful they should better explain the relevant physics- given both boundary condition at both side of the network and the geometry of the network.

We agree with the referee that indeed, if one of the network’s boundaries is force free, the polymerizing network will push this boundary without exerting any force. However, almost all mechanistic models on the role of actin in endocytosis suppose a linkage between the cytosolic part of the actin network and the endocytic coat (see Manenschijn elife 2019, Kaksonen Nature Reviews Molecular Cell Biology 2018, Wang PLOS Computational Biol 2017, Kaksonen Nature Review Molecular Cell Biology 2006 ). This linkage is typically thought to be mediated through the protein Sla2. Thus the network present at the flat plasma membrane polymerizes and pushes inside the cell the older network on the cytosolic side which is linked via the coat to the curved membrane being deformed. We modified the text of the model to clarify this point.

Minor comments:

- Ref. 22- there is no journal name
- Lines 291-292 “This is however IS not very surprising…”

These corrections were made, and we thank Reviewer #1 for his/her thorough reading.

**Reviewer #2’s comments:**

This manuscript addresses the role of mechanical stiffness of the endocytic actin network in the budding of endocytic vesicles in yeast cells. The mechanical properties of the endocytic protein machinery are a very poorly studied due to significant technical challenges in analyzing these tiny cellular structures. This question, however, is critical for mechanistic understanding of endocytosis, and for understanding other mechanical processes in cells, such as cell migration. The authors use a new and very innovative approach to tackle this question. They use in parallel an in vitro approach and an in vivo approach, and correlate the results from these two complementary approaches. The in vitro assay allows them to directly measure the mechanical stiffness of reconstituted endocytic actin patches with or without key actin filament crosslinkers. The in vivo approach reveals the endocytic functionality of the wildtype and comparable crosslinker mutant strains. The manuscript shows that crosslinker proteins increase the stiffness of the actin patch and that enough stiffness is essential for the proper budding of endocytic vesicles. The results and the approach are very interesting for the field of endocytosis, but also for the broader audience of people working on the cytoskeleton and cellular biomechanics. The manuscript is very clear and concise. The experiments are well performed and controlled. I have only very minor suggestions for the authors.

We thank Reviewer #2 for his/her positive opinion on our manuscript.

-	In the abstract use “actin crosslinkers” instead of just “crosslinkers”.

We have modified the abstract as requested.

-	Fig 1B: label the strains. There are no “black dots” in the fig 1B as stated in the legend.

 The black dot of a previous version were replaced by a dotted line, but we forgot to modify the legend accordingly. This error is now repaired.

-	Fig 4: Although the model figure is quite clear, it would be helpful to have a figure legend that briefly describes the model.

 We have added the following legend:

This model proposes that in WT cells (upper panel), the mechanical properties of actin patches (green filled dots) are optimized for efficient force transmission at the plasma membrane (in blue) and a rapid internalization of the endocytic vesicles. Subsequently to the internalization of an endocytic vesicle, the actin network disassembles and contributes to replenishing the limiting pool of actin monomers (green open dots) up to a normal level. In mutant strains, defective actin crosslinking (crosslinkers are in red) impacts the stiffness of actin networks. Force transduction to the membrane is less efficient, increasing actin patch lifetime and the proportion of failed endocytic events. Longer lifetime of actin patches for an equivalent frequency of endocytic events initiation accounts for a higher number of actin patches in these mutant cells. As the pool of actin monomers is limiting, an increased number of actin patches means that more actin is polymerized in cells and that less monomeric actin is present. Depletion of the pool of monomeric actin leads to a reduced rate of actin assembly which further fails endocytosis.
